# The U.S. Federal Menthol Ban: a Critical Step Toward Health Equity for African Americans

**DOI:** 10.1007/s40615-026-03011-0

**Published:** 2026-05-12

**Authors:** Kyrah Frank, Chanita Hughes Halbert, Trista A. Beard

**Affiliations:** 1 Department of Psychology, California State University, San Bernardino, USA; 2 Department of Population and Public Health Sciences, University of Southern California, 1845 N Soto Street, 3rd Floor, SSB 302J, Los Angeles, CA 90032 USA

**Keywords:** Tobacco-related disease, Health disparities, Menthol, Tobacco policy, Black health

## Abstract

Despite longstanding evidence of the disproportionate burden menthol cigarettes pose to Black Americans, the U.S. Food and Drug Administration’s (FDA) proposed federal ban has faced consistent delays. Menthol is associated with an increased risk of addiction, fewer quit attempts, and disproportionate rates of smoking-related illness and death among Black Americans. While states like Massachusetts and California have taken steps to implement bans, the U.S. federal government has yet to act. These delays are a public health failure that perpetuates racial health disparities. This paper explores the historical targeting of Black communities by the tobacco industry and highlights the complex responses to the potential ban from those in Black communities. Despite mixed public opinion, the evidence is clear that a federal menthol ban, combined with equitable culturally tailored cessation resources, can save lives and address disproportionate tobacco-related health disparities.

## Introduction

The continued sale of menthol cigarettes in the U.S. contributes to health disparities for Black Americans. Black Americans have one of the highest rates of smoking across racial groups at 19.4% [1]. The effects of smoking disproportionately burden the Black community; when compared to other groups, Black Americans are more likely to develop tobacco-related cancer as well as face the highest mortality burden from related illnesses (i.e., stroke, heart disease, hypertension) [2]. While tobacco use and tobacco-related health disparities are a global concern, this essay is focused strictly on the U.S. federal ban of menthol cigarettes. Menthol cigarettes are one subtype of combustible tobacco products that contain menthol, a chemical compound found in mint plants and commonly lab-synthesized and added to tobacco to produce a cooling sensation when inhaled [3]. The sensory additive is meant to reduce the harshness of smoke, which increases appeal and has led to higher rates of youth initiation and continued smoking [4]. Producers of commercial tobacco products are now multinational corporations generating tens of billions in revenue annually (e.g., Phillip Morris International, British American Tobacco, R.J. Reynolds Tobacco, Altria, Imperial Brands, et al.), and these companies dominate the cigarette market and make up what we refer to here as the “tobacco industry.”

In the U.S., a federal menthol ban was proposed in 2022 by the Food and Drug Administration (FDA) and seemed on track to be approved after the public comment period, but it never materialized [5]. Despite the failure of the ban to be federally adopted, two states have acted against the sale of menthol cigarettes, as well as hundreds of local jurisdictions. In 2020, Massachusetts became the first state to ban the sale of menthol cigarettes, and soon after, California adopted a similar ordinance against the sale of menthol cigarettes [6, 7]. Since the adoption of state-wide bans, public opinion has been divided both for and against comprehensive health policies that seek to protect the public and limit the range of tobacco products on the market. Participants in recent studies reported that they viewed a statewide ban of tobacco products as protective for the public, but also perceived a ban as inhibiting the personal freedoms of adults to use legally sold substances [6, 7]. While public opinion on the FDA’s menthol ban may swing between concern for community health and protection of personal freedoms, the delay in the federal prohibition on sales of menthol cigarettes is a health disparity issue in the U.S.

Today, over 85% of Black smokers in the U.S. use menthol cigarettes, compared to approximately 30% of white smokers [8, 9]. Black communities face unique systemic factors related to tobacco, such as the lower cost and greater availability of menthol cigarettes in their neighborhoods, which further perpetuate this disparity [10]. Also, a preference for menthol at the time of smoking initiation is linked to menthol’s ability to mask the harshness of smoking, making it easier to inhale deeply, which can lead to higher addiction rates and greater health risks [11]. A combination of systemic racism, structural inequalities, high levels of social stress, targeted advertising, and a product that masks its lethality have created a health crisis for Black Americans who smoke [1, 12]. 

The public health consequences are severe, with Black Americans experiencing disproportionately high rates of smoking-related illnesses, including heart disease, stroke, and lung cancer, from which Black men are 15% more likely to die than their White counterparts [1]. In response, the FDA proposed banning menthol in cigarettes, recognizing its role in health disparities [8]. While health experts suggest that the positive impact on health among the Black community will be significant, there remains substantial mistrust among Black Americans from a history of detrimental governmental practices and policies (i.e., policing, housing, medical research, justice system, voter suppression), which stand in parallel to the predatory practices of the tobacco industry targeting this community through advertisements, discounts, and promotions, and menthol flavoring [13]. 

## Historical Targeting of Black Communities by the Tobacco Industry

The preference for menthol cigarettes among Black Americans is rooted in a history of racially targeted marketing. Tobacco companies began aggressively marketing menthol cigarettes to Black communities in the mid-20th century, using culturally tailored advertisements, sponsorship of Black cultural events, and partnerships with Black media outlets such as *Ebony* and *Jet* [14]. These marketing strategies positioned menthol cigarettes as symbols of sophistication and community identity, contributing to their widespread adoption. The targeted advertising was successful in identifying themes of cultural relevance and symbols that resonated with Black Americans [14]. The history of predatory marketing has moved beyond featuring Black models in ads; tobacco companies place more advertisements in neighborhoods where more Black Americans live, and more commonly use price promotions to increase sales in minority neighborhoods [15]. Yet the tobacco industry has also seemingly provided economic investments to such communities; they offered jobs, sponsorships, and financial contributions of millions of dollars a year to establish community partnerships [16]. The same playbook was used to target LGBT youth and promote menthol and other tobacco products by sponsoring events such as Pride festivals, LGBT community events, fashion shows, and donations to HIV/AIDS organizations [17]. The investment is always to promote the brand and, unfortunately, the cost is always people’s health and, ultimately, their lives [2]. Combating targeted tobacco marketing with protective health policy in parallel with culturally relevant health promotion will be a key countermeasure.

Beyond the historical targeted marketing of menthol products to Black Americans, the tobacco industry has strategically used delays, blocks, and loopholes to circumvent long-awaited bans on menthol cigarettes. In 2014, the European Union Tobacco Product Directive ban was passed, criminalizing the sale of menthol cigarettes in the UK [18]. However, the ban was delayed for six years, giving the industry time to increase menthol sales. Further, the COVID-19 pandemic gave the tobacco lobbyists in the UK an opportunity to haggle for more time to comply with the ban, and subsequently, cigarette sales continues to increase [18]. In California, the tobacco industry reformulated products with synthetic cooling chemicals and rebranded lines as non-menthol to undercut the law, while seeming to comply with the flavor ban [19]. Although it could be argued that menthol cigarettes are not more dangerous than non-menthol tobacco products, the history of racial (and youth) targeting through tobacco marketing, the high prevalence of menthol use among Black smokers, and the disparate smoking-related health outcomes for Black Americans make it clear that menthol cigarettes are a health equity issue [20, 21]. Removing all menthol-flavored products and menthol additives from the market would be desirable, since these products have long been proven to have increased appeal for youth, increase early initiation, and increase long-term smoking addiction [4].

## Public Attitudes and Perceptions on a Menthol Ban

Public attitudes ahead of a federal menthol ban are diverse and at times contrary. Two recent studies [6, 7] on public opinion examined the views and perceptions on a menthol ban, and neither found a majority opinion opposing or in support of the ban. On one hand, the public was concerned that the government was overstepping and impeding personal decision-making. Some questioned the motives of the FDA and other regulators, suggesting the ban was financially motivated, politically motivated, or not implemented solely for public health concerns [6, 7]. Further, participants were concerned that a ban would unjustly target and discriminate against the Black community [6]. On the other hand, other participants reported that a ban could increase cessation, stop youth from initiating tobacco use, mitigate the targeting of people of color toward menthol products, and improve efforts in the promotion of healthy behaviors [6, 7]. A study by Czaplicki et al. [13] found that high public support for a menthol ban came from populations and communities that have been targeted by the tobacco industry (i.e., Black, Hispanic/Latino, low-income), which highlights these communities’ desire for remediation.

## Responses to the Potential Ban from Black communities

Responses from Black communities to a proposed menthol cigarette ban reveal a mix of support and concern. Research suggests that nearly half of Black smokers report they would attempt to quit if menthol were banned, significantly improving health outcomes [15, 22]. However, opposition arises from concerns about unintended consequences, including fears of racial profiling during enforcement, parallels to discriminatory policing practices, and perceived restrictions on personal freedoms [23]. These concerns are further complicated by the influence of the tobacco industry, which financially supports individual rights groups that oppose the ban [24]. This duality underscores the importance of balancing public health goals with equitable and just implementation of such policies.

In a qualitative study, authors D’Silva et al. completed in-depth interviews with Black Americans who smoke to discuss the impact a potential menthol ban could have on quit attempts, leading to improved health in the Black community [25]. After decades of predatory behavior from the tobacco industry, menthol users were aware of the harm to their health and that of their community. Black Americans who use menthol products said they knew that these products contain highly addictive properties that contribute to the disparate rates of cancer and death in their community [25]. Participants expressed concern for their health and were optimistic that a menthol ban might lead to quit attempts, which increase the odds of cessation [25]. 

A recent mixed-methods study with 213 Black Americans who smoke predicted that the likelihood of quitting would increase with a menthol ban [26]. Both men and women were surveyed and interviewed on their motivations and barriers to quitting, with health risks and cigarette costs predicting higher quit intentions, and weight-gain concerns predicting lower quit intentions. In the interviews, smokers were candid about their perceptions that menthol cigarettes were more harmful due to the additives and more addictive because of the flavoring [26]. Responses to the ban varied; while many respondents said they would attempt to quit, others said they would switch to a non-menthol product or attempt to add a menthol-like taste to their cigarettes [26]. Lastly, for long-term cessation to be successful after a ban, White et al. suggest messaging and cessation supports that are embedded in the community, that address specific barriers to quitting (e.g., weight gain), and involve the perspectives of community members who have successfully quit smoking.

## Potential Benefits of the Menthol Ban

While opinions vary on this topic, researchers are optimistic that a federal ban will affect behavior change for those struggling to quit, stating that if even a small percentage of young adult smokers quit because of the ban, it would result in a significant health gain [27]. Menthol cigarettes are associated with greater nicotine dependence and can make quitting more difficult [3]. Several studies have found equal or lower cessation rates for menthol smokers, but saw more pronounced differences for Black Americans, who had lower quit rates and higher resumption rates [28–30]. Early evidence suggests that removing these products from the market may lead to measurable public health benefits. For instance, Booras et al. reported that a statewide menthol ban in Massachusetts led to at least one quit attempt among half of their participants [6]. Menthol smokers in Canada were more likely to attempt to quit and more likely to remain quit over time, after the national menthol ban in Canada [31]. Based on an expert elicitation, a successful implementation of a federal menthol ban shows promise in reducing the health disparities among Black Americans, with a predicted 39% drop in smoking initiation [32]. While smoking rates have decreased for most racial and ethnic groups over the past decade [33], the use of menthol cigarettes has continued to increase [34]. Recent data shows an increase in menthol smoking among Hispanic populations as well, demonstrating where the federal ban could further reduce health disparities among minority populations [35]. A modeling study projected that if a menthol ban had been implemented in 2021, overall smoking rates would have declined by nearly 15% by 2026, potentially preventing over 16,000 smoking-related deaths annually [34]. 

Decreased access to menthol will be the contributing factor in increased quit attempts, as well as increasing the prevention of youth initiation [36]. A recent meta-analysis and systematic review by Mills et al. included 78 studies that provided evidence from real-world menthol bans, as well as research that asked smokers how they would respond if the U.S. menthol ban were enacted [36]. The authors found that about a quarter of menthol smokers quit in areas after a ban went into effect, demonstrating a powerful health impact. Half of smokers in the pooled data switched to a different product (non-mentholated or other flavor), or crossed into another jurisdiction to purchase menthol [36]. Local menthol bans are particularly vulnerable to cross-border purchasing, slowing the progress the bans aimed for, which further supports the need for a federal mandate to reduce loopholes and reduce smoking overall [36]. Moreover, federal legislation on menthol is the correct step forward to enacting reparative justice due to the ban’s potential to reduce tobacco-related health disparities. After decades of Black Americans being targeted by tobacco companies with specific promotions of menthol products [37], the federal menthol ban will help reduce health disparities by benefiting groups with high rates of menthol cigarette use, including Black Americans. This policy adoption could serve as a concerted effort toward reconciliation and reparations among the Black community. Regulatory action combined with culturally relevant cessation support will be imperative to reduce the smoking-related cancer burden among Black Americans.

## Tailored Cessation Support

Smoking prevalence is currently on a decline nationwide, but the rate has been more rapid in states like California and Massachusetts with statewide tobacco control programs, and slower in states with a high prevalence of smoking among older adults (> 50) [38]. Nationally, significant reductions have been driven by public health policies, increased cigarette taxes, and tobacco control programs, which vary by state. Socioeconomic factors and disparities in access to cessation resources also contribute to regional differences [39]. The decline in smoking is attributed to each state’s tobacco control programs, but Black Americans are not reaping the benefits.

Researchers suggest that each state needs culturally tailored tobacco treatment programs to increase the success of cessation [6]. Culturally specific tobacco prevention, treatment, and cessation programs tailored to Black Americans have demonstrated the potential to improve engagement and quit rates by addressing unique socio-cultural factors and community needs. For instance, interventions such as the “Freedom from Smoking” program adapt standard methods to include culturally relevant materials, facilitators from the target community, and discussions on socio-cultural barriers and marketing practices that perpetuate smoking in Black communities [40]. These approaches, rooted in frameworks like the PEN-3 model, emphasize culturally resonant messaging to counter myths about cessation therapies, highlight the health and financial benefits of quitting, and encourage readiness to quit [41, 42]. In a meta-analysis of 15 clinical trials of tobacco cessation treatments in samples composed primarily of African American participants, the authors found the most effective cessation outcomes were observed for interventions that combined behavioral counseling with pharmacologic aids (e.g., nicotine replacement therapy or other medications). Although culturally tailored cessation interventions did not significantly improve abstinence rates, the authors suggest that such tailoring may play an important role in reducing attrition and helping participants remain engaged throughout the cessation program [43]. 

While culturally-tailored cessation resources are an evidence-based recommendation, they are not a one-size-fits-all solution. Webb found that while Black smokers expressed a preference for culturally specific self-help and cessation education materials, the abstinence outcomes differed among groups in the Black community [44]. Black Americans are not a monolith, but a heterogeneous group with a wide variety of intragroup differences in acculturation. Ethnic identity can meaningfully shape how people respond, so assessing and matching interventions to acculturation (and possibly other cultural factors) is critical for both engagement and outcomes [44]. While progress on a federal menthol ban remains stalled, this period provides an opportunity for the research community to strengthen and evaluate cessation interventions and tobacco education efforts. Developing these strategies in partnership with Black community members will help ensure they are effective and ready to be implemented when the policy environment improves.

Statewide tobacco control programs have been effective at reducing menthol sales and lowering smoking prevalence, which impacts health disparities, but state bans do not provide a comprehensive health policy to protect all Black Americans. We would argue that all Black Americans deserve special attention to account for years of targeted marketing at the cost of their health. To address decades of health segregation, state and federal health policymakers should act on the FDA menthol ban and move resources toward health promotion, tobacco prevention education, and cessation programs culturally tailored to Black Americans who experience a higher burden of tobacco-related disease and illness.

## Conclusion

Black Americans have experienced a disproportionate cancer burden, and, just as with other protective health policies (i.e., seatbelts, public building smoking bans), a federal ban on menthol products will lead to a decrease in smoking-related illness and disease among Black smokers. While some current menthol users are concerned with government limitations on individual rights [6, 7], there is broad community support for a federal menthol ban [13]. Despite the swing of public perceptions and opinions, the health benefits that will follow a ban are of the utmost importance. A U.S. ban on menthol has the potential to decrease overall smoking rates and prevent thousands of smoking-related illnesses and deaths [34]. Further, a ban could increase quit attempts, reduce youth initiation, and ultimately increase community-wide health and overall quality of life [25]. For a federal menthol ban to be most effective, implementation must include equitable enforcement (targeting tobacco companies and retailers and not criminalizing persons who smoke for use or possession) and provide a variety of culturally tailored resources for cessation support that are built in partnership with community health advocates, ensuring that impacted individuals have the tools to quit successfully. The federal menthol ban languished for years in the public comment period with no action taken, and was immediately withdrawn under the Trump Administration [45]. The evidence of harm to the Black community caused by menthol products is well-documented. Now we call upon public health researchers, health equity leaders, clinician-scientists, and policymakers to renew their commitment to reducing access to flavored products and to building tobacco-free communities.

## Data Availability

Not applicable.
